# Patterns of Lynx Predation at the Interface between Protected Areas and Multi-Use Landscapes in Central Europe

**DOI:** 10.1371/journal.pone.0138139

**Published:** 2015-09-17

**Authors:** Elisa Belotti, Nicole Weder, Luděk Bufka, Arne Kaldhusdal, Helmut Küchenhoff, Heidi Seibold, Benno Woelfing, Marco Heurich

**Affiliations:** 1 Faculty of Forestry and Wood Sciences, Czech University of Life Sciences Prague, Kamýcká 1176, 16521 Prague 6, Czech Republic; 2 Department of Research and Nature Protection, Šumava National Park Administration, Sušická 399, 34192 Kašperské Hory, Czech Republic; 3 Department of Research and Documentation, Bavarian Forest National Park, Freyunger Str. 2, 94481 Grafenau, Germany; 4 Department of Statistics, LMU Munich, Ludwigstraße 33, 80539 Munich, Germany; 5 Wildlife Ecology and Management, University of Freiburg, Faculty of Environment and Natural Resources, Tennenbacher Straße 4, 79106 Freiburg, Germany; University of Sydney, AUSTRALIA

## Abstract

In Central Europe, protected areas are too small to ensure survival of populations of large carnivores. In the surrounding areas, these species are often persecuted due to competition with game hunters. Therefore, understanding how predation intensity varies spatio-temporally across areas with different levels of protection is fundamental. We investigated the predation patterns of Eurasian lynx (*Lynx lynx*) on roe deer (*Capreolus capreolus*) and red deer (*Cervus elaphus*) in both protected areas and multi-use landscapes of the Bohemian Forest Ecosystem. Based on 359 roe and red deer killed by 10 GPS-collared lynx, we calculated the species-specific annual kill rates and tested for effects of season and lynx age, sex and reproductive status. Because roe and red deer in the study area concentrate in unprotected lowlands during winter, we modeled spatial distribution of kills separately for summer and winter and calculated-the probability of a deer killed by lynx and-the expected number of kills for areas with different levels of protection. Significantly more roe deer (46.05–74.71/year/individual lynx) were killed than red deer (1.57–9.63/year/individual lynx), more deer were killed in winter than in summer, and lynx family groups had higher annual kill rates than adult male, single adult female and subadult female lynx. In winter the probability of a deer killed and the expected number of kills were higher outside the most protected part of the study area than inside; in summer, this probability did not differ between areas, and the expected number of kills was slightly larger inside than outside the most protected part of the study area. This indicates that the intensity of lynx predation in the unprotected part of the Bohemian Forest Ecosystem increases in winter, thus mitigation of conflicts in these areas should be included as a priority in the lynx conservation strategy.

## Introduction

After almost two centuries of declines and extinctions, large carnivores during recent decades have re-colonized large parts of their historical ranges in Europe and North America [1, 2]. In order to ensure their survival, most European countries have accorded legal protection to these species and have implemented several reintroduction programs [3]. In this context, the presence of a net of protected areas in which specific protection measures can be fully implemented likely play a role as source areas for populations of large carnivores [4]. In Europe, protected areas are more numerous than in any other region in the world [5]. However, most of these areas are small [6], with 90% not even reaching 10 km^2^ [7]. Given that large carnivores are territorial species with huge spatial requirements (e.g., [8]), most of these protected areas are not even large enough to encompass the territories of single specimens. Therefore, the predators generally have to expand into the surrounding multi-use, human-modified landscapes, where their probability of persistence is directly linked to their chances to coexist with human activities and be accepted by people [1, 9]. European large carnivores have proved adapt to tolerate even relatively high levels of human activities (e.g., [1, 10]), but their presence can interfere with game management and livestock farming [11, 12], resulting in human-carnivore conflicts. This has already led to the persecution of these predators in the past [11, 13] and to date still frequently leads to illegal killings, which represents one of the main threats for their long-term survival [1, 4, 11, 12]. To adequately manage these conflicts, a deeper scientific understanding of the mechanisms determining the patterns of predation by a given predator species on its prey species under different ecological conditions is required [1, 14].

The Eurasian lynx (*Lynx lynx*, hereafter: lynx) is a solitary, large stalking predator that inhabits Central, Eastern and Northern Europe with several distinct populations [15], most of which are small and isolated [3]. According to the most recent information gathered throughout Europe, lynx predation on livestock can cause relevant problems only in Northern Scandinavia [3, 16, 17], and a range of prevention and compensation measures have already been adopted by most European countries to reduce local conflicts with livestock farmers [3]. However, in all countries and especially in those areas hosting reintroduced lynx populations, a major problem is low acceptance by hunters that compete with lynx in ungulate hunting [3, 18, 19].

The European distribution of lynx largely overlaps with that of the most widespread European ungulate, the roe deer (*Capreolus capreolus*) [20], and, where both species occur together, roe deer is the main prey species of lynx (e.g., [21, 22]). The importance of other ungulate species as alternative prey, namely red deer (*Cervus elaphus*) [14, 21, 23], chamois (*Rupicapra rupicapra*) [24, 25], and reindeer (*Rangifer tarandus*) [26] varies according to their distribution across Europe.

The quantitative aspect of lynx predation on its ungulate prey, generally expressed as per-capita kill rates, has already been investigated in several European areas, namely Scandinavia [14; 26–28], Poland [22, 23], Dinaric Mountain Range [29], Swiss Alps [30, 31], and Swiss Jura Mountains [24, 25, 32]. These studies found that lynx predation rates on ungulate prey can be modulated by several factors, such as the sex, age, reproductive status, and preferences of the individual predator [26, 27, 29]; predator population status (i.e., recolonizing or established [30]); availability and distribution of a given prey species and of alternative prey species (e.g., [14]); presence of scavengers and intensity of their activity [33, 34]; and climate and winter harshness [27]. Although the relative importance of these factors varies from region to region, in the case of roe deer prey, all studies from Central Europe [23, 25, 29, 30] report similar mean values of lynx predation rates.

Although red deer is the second most important lynx prey species in several areas [21], only two studies, from Scandinavia [14] and Poland [23], have dealt with lynx kill rates of this species. Even though these studies revealed very different average kill rates, a common result of both studies was that the per-capita kill rates on red deer widely varied among lynx of different sex and reproductive status.

Besides the quantitative aspect, some studies [14, 23, 26, 29] have also considered the “seasonal aspect” of predation by lynx, i.e. potential changes in lynx predation rates throughout the year. These studies obtained contrasting results: kill rates were higher in winter than in summer in most of Scandinavia [14, 26], the opposite was observed in the Central European Dinaric Mountains [29] and no substantial seasonal differences in kill rates were found in Poland and Southern Sweden [23, 35].

Finally, another important aspect of lynx predation that has so far received little attention is the “spatio-temporal aspect”, i.e., the distribution of killed prey throughout the landscape and the potential changes in this distribution during the year. Lynx may not kill their prey evenly throughout their home ranges and the predation probability may be concentrated in particular sub-areas, e.g., those characterized by higher prey densities or by habitat features that increase prey vulnerability [26, 36]. Furthermore, red and roe deer typically seasonally migrate and modify their grouping behavior in most mountainous European regions [37–40], concentrating at low elevations with milder snow conditions in winter and spreading again toward higher elevations to exploit richer foraging condition in summer [41, 42]. As a consequence of these migrations, also the spatial pattern of lynx predation may change throughout the year. Because several European protected areas are located in mountainous lands [7, 43], this may lead to a substantial seasonal increase in lynx predation outside of these areas, where sport hunting is widely practiced. Therefore, taking this last point into consideration can greatly help identifying where and when conflicts between lynx and hunters may arise.

In the Bohemian Forest Ecosystem, a central-European region encompassing large protected areas and multi-use landscapes, we analyzed the spatio-temporal, together with the quantitative and seasonal aspects of lynx predation on the main prey species in the area, roe and red deer [44]. In particular, we tested the following hypotheses:

1. Following the seasonal changes in deer distribution, predation by lynx in winter will be more spatially concentrated and will be highest outside of the National Parks (that are the areas where the highest level of protection is ensured and that include mostly mountainous areas), whereas predation by lynx in summer will be more spread and will not differ inside and outside the National Parks;
2. Average kill rates for roe and red deer will be similar to those found elsewhere in Central Europe and higher than those found in most of Scandinavia, given the generally lower densities of deer and the higher importance of alternative prey in Scandinavia [26, 28];
3. Kill rates for both red and roe deer will vary between seasons (summer, winter) and between lynx of different sex, age, and reproductive status.

## Material and Methods

### Study area

The Bohemian Forest Ecosystem (hereafter: BFE) is a forested low mountain range located along the German–Czech border and is the largest contiguous region of strictly protected woodlands in Central Europe. It extends from a height of 600 m above sea level (a.s.l.) in the valleys to 1,456 m a.s.l. on the ridge lines and is characterized by long (5–8 months), cold, and snowy winters, followed by relatively warm summers. The average annual temperature lies between 6.7°C in the valleys and 3.9°C at high elevations. Annual precipitation between 1,085 and 1,860 mm is common [45]. In winter, the average snow depth is 40–60 cm in valleys and 100–120 cm at higher altitudes, where maximum values of about 3 m can be reached. At higher elevations, the dominant tree species is Norway spruce (*Picea abies*), accompanied by mountain ash (*Sorbus aucuparia*), while lower ranges are characterized by Norway spruce, European beech (*Fagus sylvatica*), and silver fir (*Abies alba*) [46]. The Bavarian Forest National Park (240 km^2^; 49°3'19"N, 13°12'9"E) on the German side and the Šumava National Park (690 km^2^; 49°7'0"N, 13°36'0"E) on the Czech side of the national border represent the core of this area, where the most rigorous measures of nature protection are applied. On the Czech side, this core area is surrounded by the Šumava Protected Landscape Area (996 km^2^, 49°11′52″N, 13°14′25″E), where a wider range of human activities is permitted, but special attention is still given to nature conservation.

In the BFE as in most of Europe, the Eurasian lynx became extinct in the mid 19th century [47]. Its reintroduction began in Bavaria in the early 1970s, with the release of an uncertain number of animals [48]. Between 1982 and 1989, 18 lynx were released on the Czech side of the Šumava Mountains [49]. During the 1990s, the Bohemian–Bavarian lynx population increased significantly [47], but during the following years it declined. At present, this lynx population is stagnant [4], with estimated densities ranging from 0.4 to 0.9 independent lynx/100 km^2^ for the core area [50].

Regarding the density of the main prey of lynx on the German side, the red deer density is 1.56 animals/km^2^, as estimated via coordinated counts at feeding stations in the Bavarian Forest National Park during winter. A minimum roe deer density of 1.61 animals/km^2^ (1.1–2.3) was determined by distance sampling with thermal cameras inside the Bavarian Forest National Park in spring [51]; the roe deer density is generally higher outside of this strictly protected area [52]. Although such precise data are not available for the Czech side, some studies indicate that roe deer and red deer densities in the Šumava National Park are at least twice as high as those reported for the Bavarian Forest National Park, and roe deer densities are even higher in the Šumava Protected Landscape Area and its closest unprotected surroundings [52]. On both sides of the national border, a large proportion of both roe and red deer populations seasonally migrates along an altitudinal gradient, concentrating in the valleys during winter time [41, 53].

Inside the two national parks, hunting of roe deer is completely banned. Red deer are regulated during established hunting seasons by a limited number of qualified national park employees, merely as a measure of population control because wolves (*Canis lupus*), i.e., the main predators of red deer, are absent from the BFE [52, 54]. Outside of national parks, sport hunting of both deer species is practiced during specific hunting seasons and according to defined hunting plans [52]. In most of the lowland areas surrounding the national parks, red deer is intensively shot in order to limit damages to forestry. On the German side, the absence of the species from these areas is even established by law [55, 56].

**Fig 1:**
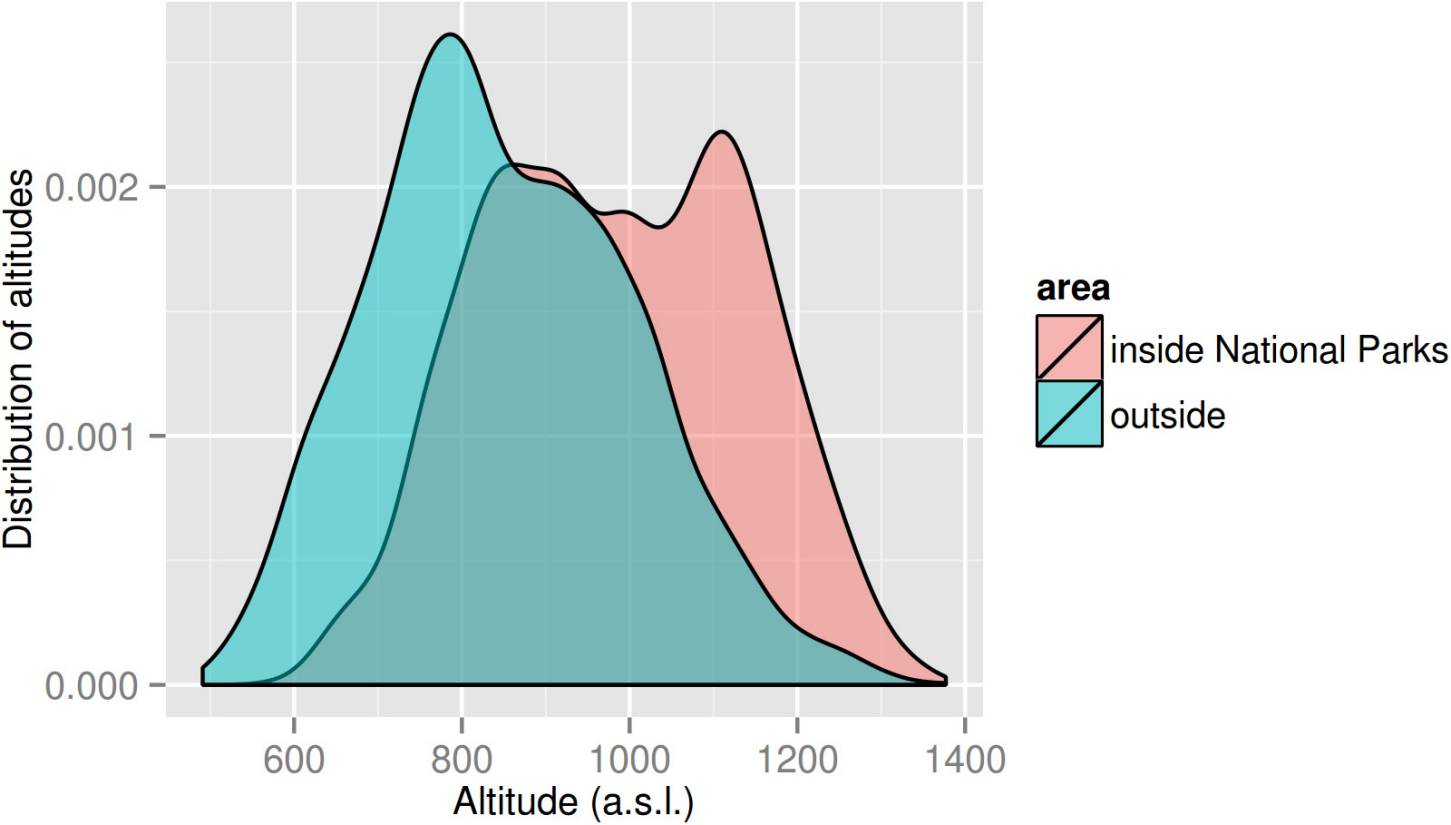
Distribution of altitudes within the study area, inside and outside the two national parks.

In our study, the study area was defined as the combination of the 95% Minimum Convex Polygons (MCP) home ranges of all monitored lynx. This area measures about 1,623 km^2^ and includes about 2/3 of the Šumava National Park and Bavarian Forest National Park (i.e., 46% of the study area), half of the Šumava Protected Landscape Area (21%), and a wide belt of unprotected surroundings (33%). While the area of the two national parks includes the main mountain ridge, most of the valleys are located in the Šumava Protected Landscape Area and the unprotected surroundings (see Fig 1).

### Lynx telemetry

Between March 2005 and March 2012, ten lynx (six males and four females, Table 1) were captured, using walk-through box traps either at a kill site or at known lynx trails (for a detailed description of the handling protocol, see Heurich [57]). All lynx were immobilized with 1–1.2 mL of “Hellabrunn mixture” (400 mg ketamine and 500 mg xylazine) and fitted with GPS-GSM collars (VECTRONIC Aerospace, Berlin, Germany). One male and one female were caught as subadults (< 2 years old), and one female was caught as a juvenile (< 1 year old). All three animals became adult (> 2 years old) before the end of their monitoring periods. All females reproduced at least once (litter size = one or two kittens) during their monitoring periods. Collars stored two positions daily, at midnight (00:00 Central European Time: CET = UTC + 1) and midday (12:00). For a one-month period in each season of the year, two additional positions per day were recorded at dusk and dawn (i.e., the periods of highest lynx activity [58, 59]). Finally, once every second week, collars recorded one GPS position per hour from 15:00 to 7:00 of the following day. Collars were also equipped with a VHF transmitter, which allowed us to locate the collared animal for about one year after the collar GPS-GSM device had stopped working. We identified the locations where lynx may have killed a prey of medium to large size as clusters of night GPS and/or VHF positions (see Podolski et al. [58] for further details). Such locations were searched in the field with the help of a GPS-receiver and dogs. In addition, in order to minimize the possibility to miss ungulate prey that may be abandoned by lynx soon after having been killed, we additionally searched for prey remains at about 300 randomly chosen single GPS positions recorded during the period from dusk to dawn.

**Table 1:** Overview information about the ten GPS-collared lynx: lynx status (determined based on the sex, age and on the presence and number of kittens; status of all females varied throughout the monitoring period), length of monitoring period, number of confirmed killed prey and of roe deer and red deer kills, and home range size (95% MCP).

Lynx ID	Lynx status			Date of capture	Monitoring period (GPS+VHF telemetry) [days]	Number of kills	Roe deer	Red deer		95% MCP [km2]
	Sex	Age	Kittens							
**M1**	m	a		07/03/05	509	9	6	3		532
**F1**	f	a	1; 2	17/01/07	724	22	20	0		166
**M1**	m	a		12/11/08	460	38	28	9		532
**M2**	m	a		27/03/10	103	13	11	1		225
**M3**	m	a		11/03/10	378	38	25	11		389
**F2**	f	a	2	17/03/10	606	55	52	1		145
**F3**	f	j, s, a	2	17/03/10	870	70	63	6		101
**M4**	m	s, a		15/01/11	473	42	20	22		462
**F4**	f	s, a	2	27/02/11	399	36	31	2		77
**M5**	m	a		22/03/11	590	48	35	10		257
**M6**	m	a		10/03/12	549	39	36	3		520
** **					**TOTAL**	**410**	**327**	**68**	**ø Female**	**122**
									**ø Male**	**432**

Male M1 was monitored during two non-sequential time periods; therefore, information for each monitoring period is reported separately. “ø Females” and “ø Males” = mean home range size of female and male lynx, respectively; “m” = male; “f” = female; “a” = adult; “s” = subadult; “j” = juvenile.

### Ethics statement

The handling protocol was approved by Ethics Committee of the Government of Upper Bavaria and the Czech Central Commission for Animal Welfare and fulfils their ethical requirements for research on wild animals (permit number: 55.2-1-54-2532-82-10). Both mentioned institutions specifically approved this study. In addition, permits for wild animal capture were obtained from the Government of Lower Bavaria (permit number: 55.1–8621.1–57), the Czech Central Commission for Animal Welfare (permit numbers: 44048/2008-17210, 44048/2008-10001) and the Czech Ministry of Environment (permit number: 41584/ENV/10-1643/620/10-PP8). Searching for prey in the field was allowed by the Administrations of the Šumava National Park and Bavarian Forest National Park and by landowners outside of the national parks.

### Kill series

A total of 410 prey remains were found in the field (Table 1 and S1 Dataset), of which 79.76% were roe deer, 16.58% red deer, 0.49% wild boar (*Sus scrofa*), 0.98% red fox (*Vulpes vulpes*), and 2.19% brown hare (*Lepus europaeus*). For this study, we took into account only roe deer and red deer kills. In addition, kills made by family groups with one kitten, juvenile lynx, and subadult male lynx were not considered because of insufficient sample size (n = 4, 2, and 11 killed prey, respectively). For each deer prey killed by adult males, single adult females, subadult females and family groups with two kittens we calculated:

1. The handling time, i.e. the time that lynx spent at a prey [60], calculated as the number of nights a lynx visited the same killed prey, according to its GPS and/or VHF positions.
2. The searching time, i.e., the time between when a lynx left its prey until it killed the next prey [23]. In order to reduce the possibility of introducing bias to a minimum and only when calculating searching times, we also took “virtual kills” into consideration, that is, all cases (n = 173) in which a lynx seemed to have a killed prey according to GPS position patterns, but prey remains could not be found in the field [27].
3. The prey time, i.e., the sum of searching time and handling time.

We further excluded all prey for which prey time could not be calculated due to missing data from the lynx GPS collars, and we obtained a final deer prey dataset consisting of 359 lynx kills (Table 2).

**Table 2:** Number of found roe and red deer killed by lynx of each “lynx status” during each season that could be used for all calculations.

Lynx status		Roe deer	Red deer	Total
**Adult male**	Total	151 (75.1%)	50 (24.9%)	201 (100%)
	Summer	109	25	
	Winter	42	25	
**Adult female**	Total	48 (92.3%)	4 (7.7%)	52 (100%)
	Summer	28	0	
	Winter	20	4	
**Subadult female**	Total	44 (95.7%)	2 (4.3%)	46 (100%)
	Summer	33	0	
	Winter	11	2	
**Family group (2 kittens)** *	Total	58 (96.7%)	2 (3.3%)	60 (100%)
	Summer	42	1	
	Winter	16	1	

* The lynx status named “family groups” included all adult female lynx for the periods of time in which they were together with their (2) kittens.

We took the possible effect of season into consideration by dividing the year into “winter” (1 November to 31 March) and “summer” (1 April to 31 October). For both periods, in order to account for possible variations related to age, sex, and reproductive status of the lynx, we calculated handling, searching, and prey time separately for each considered lynx status.

Finally, based on the “prey series”, i.e., the series of all prey remains that were found in the field, of each individual lynx, we calculated the percentage shares of roe deer and red deer prey for each considered lynx status. These percentages were then used to determine (1) the actual prey species-specific lynx per-capita annual kill rates and (2) the (species-specific) number of deer prey killed by one lynx in one year per area unit (i.e., 1 km^2^, see below).

### Statistical analysis

#### Predation rate

In order to model the timespan between consecutive kills and hence the predation rates, an accelerated failure time (AFT) model was fitted [61]. In the AFT model, the time until a given event occurs is used as outcome, and each event is generically referred to as failure [62]. In our case, the outcome is the prey time as defined above, and the event/failure is the subsequent kill. To limit the risk of overlooking potential unidentified kills, the prey time was censored. The threshold values for censoring were chosen taking the natural behavior of lynx into account (e.g., behavioral changes of females during the denning period [60]): prey times longer than nine days for roe deer (n = 60) and longer than 12 days for red deer (n = 17) were not considered as an event.

We used the AFT model to test for effects of lynx sex, age and females’ reproductive status (“lynx status”: adult male/subadult female/single adult female/family group), prey species (roe deer/red deer) and season (summer/winter) on each of the following dependent variables: (1) prey time, (2) handling time, and (3) searching time. Second-order interactions additional to the main effects did not improve any of the models, i.e., the p-values of likelihood ratio tests comparing models with interactions to the respective main effects models were larger than 0.05. Therefore, interactions were excluded from further calculations. In order to ensure that no systematic difference between the years of the study period influenced our results, we initially included the year as covariate in all analyses. Its effect was negligible, therefore this covariate was not included in the final models. We regarded lynx identity as a random effect by incorporating a frailty term in each of these models [63, 64]. As models including the frailty term gave results very similar to those not including it, the factor accounting for lynx identity was excluded from further calculations, as well. We conducted Tukey's all-pair comparisons to investigate differences between the different lynx statuses [65]. Note that this is a testing procedure that accounts for multiple testing and hence p-values are in general higher than uncorrected p-values.

To predict the annual and seasonal predation rates, we used the AFT model, followed by bootstrapping, to generate medians and confidence intervals [66]. The interims of consecutive kills were added up in each bootstrap step until they were approximately at one year (about 365 days). For each combination of variables, 5,000 estimates for the number of kills per year were obtained and thus an empirical distribution of the estimates. To investigate the effect of season, medians and confidence intervals were also calculated with 182.5 days (i.e., 365/2). The actual annual predation rates of an average lynx of the Bohemian–Bavarian lynx population was estimated using the percentage of red deer and roe deer in the kill series of each “lynx status”:

$$annual\;predation\;rate\;=\;365days\;\cdot \;\left(\frac{percentage\;of\;deer}{100}\right)\;/\;predicted\;prey\;time$$

To obtain the predation rates per area, the annual predation rates were divided by the average home range sizes (MCP 95%) of male and female lynx living in the BFE (Table 1). All computations and statistical analyses were run in R software version 2.15.2 [67], using survival [68], plyr [69], and multicomp [70] packages.

#### Spatio-temporal variation of predation risk

To investigate how predation events were actually distributed throughout the study area and how this distribution changed throughout the year, the annual joint kill rates of roe and red deer in winter and summer were modeled using a two-stage generalized additive model [71]. Kill observations from an area consisting of 6,740 quadrants of 500x500m were modeled using the following covariates: proportion of forest cover, mean altitude a.s.l., and distance (in m) to the closest area of civilization (as defined by CORINE category 112 [72]) of each quadrant. To account for the spatial distribution of kills, of the considered covariates, and of any other spatially distributed covariates that could not be taken explicitly into account, we also included the geographic coordinates of the centroids of each quadrant. The influence of all covariates was modeled non-parametrically by means of splines [71]. Because of zero inflation (kills were observed in 2.14% of the quadrants in summer and in 2.80% in winter), we first modeled the probability to have at least one kill for each quadrant using a logistic model. Using these results, we then modeled the conditional expected number of kills in each quadrant for which at least one kill had been predicted (see S1 Appendix for further details). Note that the distance to the closest area of civilization was only used in the first step. Combining the two models, the expected number of kills in each quadrant was estimated as the conditional expected number of kills weighted with the estimated probability to observe a kill in that quadrant. In this way, we obtained a smooth distribution of kills in the entire study area. The models were fitted using the R package mgcv [71]. It is worth remarking that these estimates are unitless indicators of predation risk, whose main purpose is to identify areas with higher/lower relative risk, and therefore should not be interpreted in absolute terms (i.e., literally expected number of kills per quadrant).

Finally, the model was used to estimate the probability of observing one or more roe or red deer killed by lynx for quadrants located inside and outside of the national parks, i.e., in areas where commercial and sport hunting are banned and are widely practiced, respectively. Probabilities were estimated separately for winter and summer. As these estimates result from deterministic statistical models, we recommend only comparing them qualitatively.

## Results

### Predation rate

According to our AFT model, prey time was significantly influenced by prey species, season and “lynx status” (Table 3). The period between consecutive kills of red deer prey was much longer than that of roe deer prey (z = -5.804; p < 0.001; Tables 3 and 4). The prey time was significantly shorter in winter than in summer (z = -3.296; p < 0.001; Tables 3 and 4). According to Tukey's all-pair comparisons family groups had a significantly shorter prey time than single adult females (estimated difference = 0.386; p = 0.002), subadult females (estimated difference = 0.300; p = 0.038), and adult males (estimated difference = 0.228; p = 0.039). No significant difference was found between adult males and single adult females (estimated difference = 0.157; p = 0.319), adult males and subadult females (estimated difference = 0.071; p = 0.878), and single adult females and subadult females (estimated difference = 0.086; p = 0.883).

**Table 3:** Results of the accelerated failure time (AFT) models, with predictions of the effects on prey time, handling time, and searching time of “lynx status”, prey species, and season.

	Prey time				Handling time				Searching time			
	Effect	± S.E.	Z-value	p-value	Effect	± S.E.	Z-value	p-value	Effect	± S.E.	Z-value	p-value
**Intercept**	*2*.*516*	*0*.*125*	*20*.*210*	*<0*.*001*	*1*.*990*	*0*.*124*	*15*.*950*	*<0*.*001*	*1*.*794*	*0*.*387*	*4*.*640*	*<0*.*001*
**Lynx_status adult male**	-0.157	0.093	-1.696	0.090	*-0*.*204*	*0*.*093*	*-2*.*200*	*0*.*028*	-0.112	0.288	-0.389	0.697
**Lynx_status family group**	*-0*.*386*	*0*.*110*	*-3*.*501*	*<0*.*001*	*-0*.*393*	*0*.*111*	*-3*.*550*	*<0*.*001*	*-0*.*788*	*0*.*342*	*-2*.*308*	*0*.*021*
**Lynx_status subadult female**	-0.086	0.118	-0.729	0.466	-0.128	0.119	-1.080	0.280	0.078	0.366	0.213	0.831
**Prey_species roe deer**	*-0*.*518*	*0*.*089*	*-5*.*804*	*<0*.*001*	*-0*.*512*	*0*.*090*	*-5*.*720*	*<0*.*001*	*-0*.*699*	*0*.*277*	*-2*.*526*	*0*.*015*
**Season winter**	*-0*.*219*	*0*.*066*	*-3*.*296*	*<0*.*001*	*-0*.*265*	*0*.*067*	*-3*.*960*	*<0*.*001*	-0.268	0.206	-1.302	0.193

S.E. = standard error. Variables that proved significant in a given model (p-value < 0.05) are reported in *italics* for the given model.

**Table 4:** Prey time, handling time, searching time, predicted annual predation rates, actual annual predation rates and annual predation rates per km^2^ obtained for each “lynx status” and deer prey type using accelerated failure time (AFT) models, bootstrap for confidence intervals and percentage of each prey type.

Lynx status	Prey species	Prey time [days ± S.E.]	Handling time [days ± S.E.]	Searching time [days ± S.E.]	Predicted annual predation rate (95% confidence interval)			% of given prey type on kill series	Actual annual predation rate	Annual predation rate per km^2^
					Lower bound	Estimate	Upper bound			
Adult male	roe deer	5.95 ± 0.28	3.34 ± 0.16	2.49 ± 0.36	44	53	61	75.12	46.05	0.11
Adult female	roe deer	6.71 ± 0.56	3.90 ± 0.33	2.66 ± 0.67	39	46	53	92.31	50.22	0.41
Subadult female	roe deer	6.40 ± 0.56	3.61 ± 0.32	3.01 ± 0.80	40	49	57	95.65	54.56	0.45
Family group	roe deer	4.73 ± 0.35	2.76 ± 0.21	1.26 ± 0.29	55	66	77	96.72	74.71	0.61
Adult male	red deer	9.43 ± 0.75	5.19 ± 0.42	4.66 ± 1.13	27	34	41	24.88	9.63	0.022
Adult female	red deer	10.63 ± 1.25	6.07 ± 0.72	4.97 ± 1.78	23	30	37	7.69	2.64	0.022
Subadult female	red deer	10.14 ± 1.24	5.61 ± 0.70	5.64 ± 2.10	24	31	38	4.35	1.57	0.013
Family group	red deer	7.48 ± 0.86	4.29 ± 0.50	2.36 ± 0.82	33	41	49	3.28	1.60	0.013

S.E. = standard error. Predicted annual predation rates are reported with bootstrap 95% percentile intervals (2.5%, 97.5%).

The AFT model for handling time gave similar results (Table 3). Lynx fed on a red deer significantly longer than on a roe deer (z = -5.720; p < 0.001; Tables 3 and 4), and the handling time was significantly shorter in winter than in summer (z = -3.960; p < 0.001; Tables 3 and 4). Family groups had a significantly shorter handling time than single adult females (estimated difference = 0.393; p = 0.002). No further significant differences between lynx statuses were observed.

The AFT model for searching time showed a significant influence of prey species: the searching time for roe deer was shorter than for red deer (z = -2.526; p = 0.012; Tables 3 and 4). Concerning the searching time no significant differences between lynx statuses were found in Tukey's all-pair comparisons, but family groups tended to have a shorter searching time than adult males (estimated difference = 0.676; p = 0.053).

The predicted mean prey time, handling time, and searching time for each “lynx status” and for each deer prey species (Table 4) are only theoretical values that would be valid if lynx preyed either exclusively on roe deer or exclusively on red deer. Based on these predicted prey times, the predicted annual predation rate ranged from 53 roe deer or 34 red deer for an adult male lynx to 66 roe deer or 41 red deer for family groups (Table 4). For all lynx statuses, predicted predation rates were lower in summer than in winter (S1 Table).

In the case of roe deer prey, family groups had the highest actual annual predation rate (74.71 individuals/year, Table 4), and killed 1.6 times more roe deer than adult males (46.05 individuals/year, Table 4). The differences in the annual predation rates per km^2^ among lynx belonging to different lynx statuses were much larger, with family groups killing almost 6 times more roe deer/year/km^2^ than adult males (0.61 and 0.11 individuals/year/km^2^, respectively, Table 4).

In the case of red deer prey, adult males had the highest annual predation rate (9.63 individuals/year, Table 4), and killed approximately 6 times more red deer than subadult females (1.57 individuals/year, Table 4). The differences in the annual predation rates per km^2^ among lynx statuses were much smaller, with adult males and single adult females killing almost twice as many red deer/year/km^2^ than family groups and subadult females (0.022 and 0.013 individuals/year/km^2^, respectively, Table 4).

### Spatio-temporal variation of predation risk

**Fig 2:**
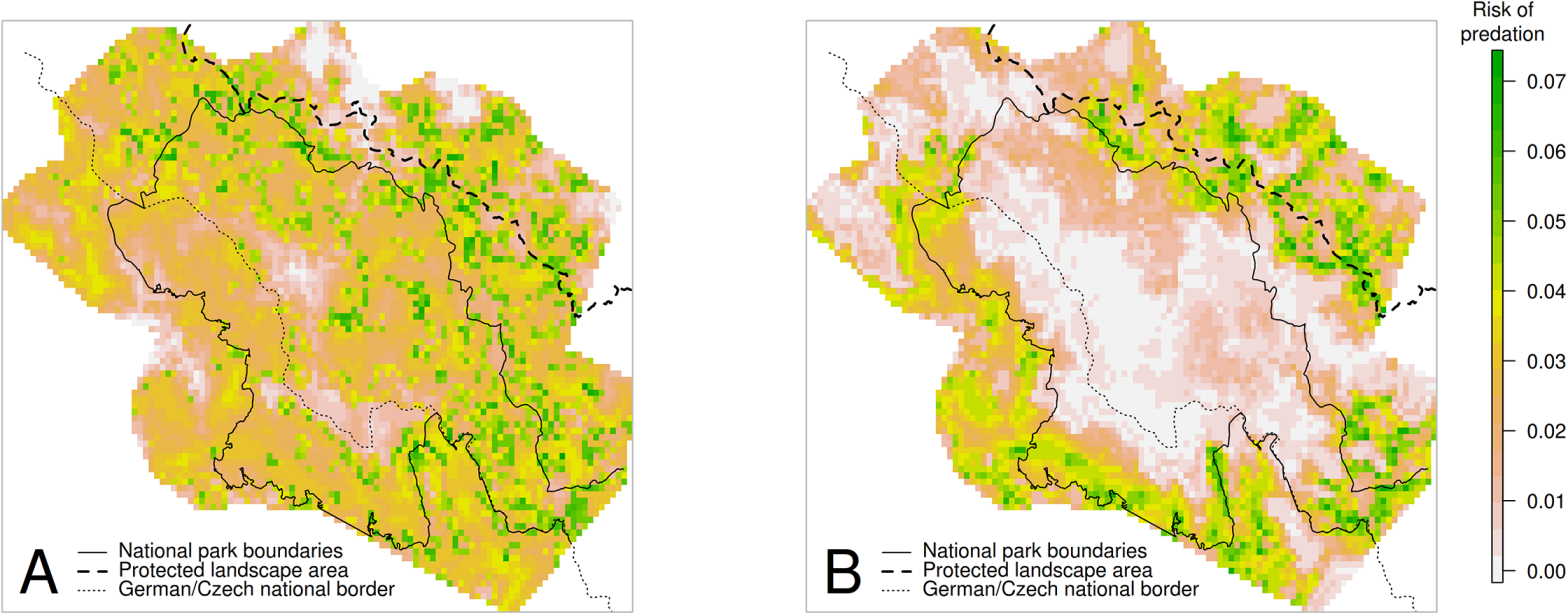
Partial predation risk for red and roe deer in summer (A) and winter (B). Estimates were obtained on the basis of a two-stage generalized additive model accounting for forest cover, altitude a.s.l., distance to civilization and the spatial alignment of the quadrants. The spatial effect was excluded from the partial risk to emphasize the spatial distribution of the effect of the biotic and abiotic factors which were explicitly included in the model. Solid line represents the borders of the two national parks; dashed line on the Czech side represents the border between the Šumava Protected Landscape Area and its unprotected surroundings. The modeled area was defined as the combination of the 95% MCPs of all collared lynx.

A spatial shift in the distributions of the kill risk, i.e., expected kills per km^2^ according to our two-stage generalized additive model, was clearly recognizable from summer (Fig 2A) to winter (Fig 2B). In summer, the predation risk was evenly distributed, whereas in winter, it was more concentrated in lower areas, outside the national parks (Pearson’s correlation between altitude and estimated predation risk of 0.1033 in summer and -0.5930 in winter).

**Fig 3:**
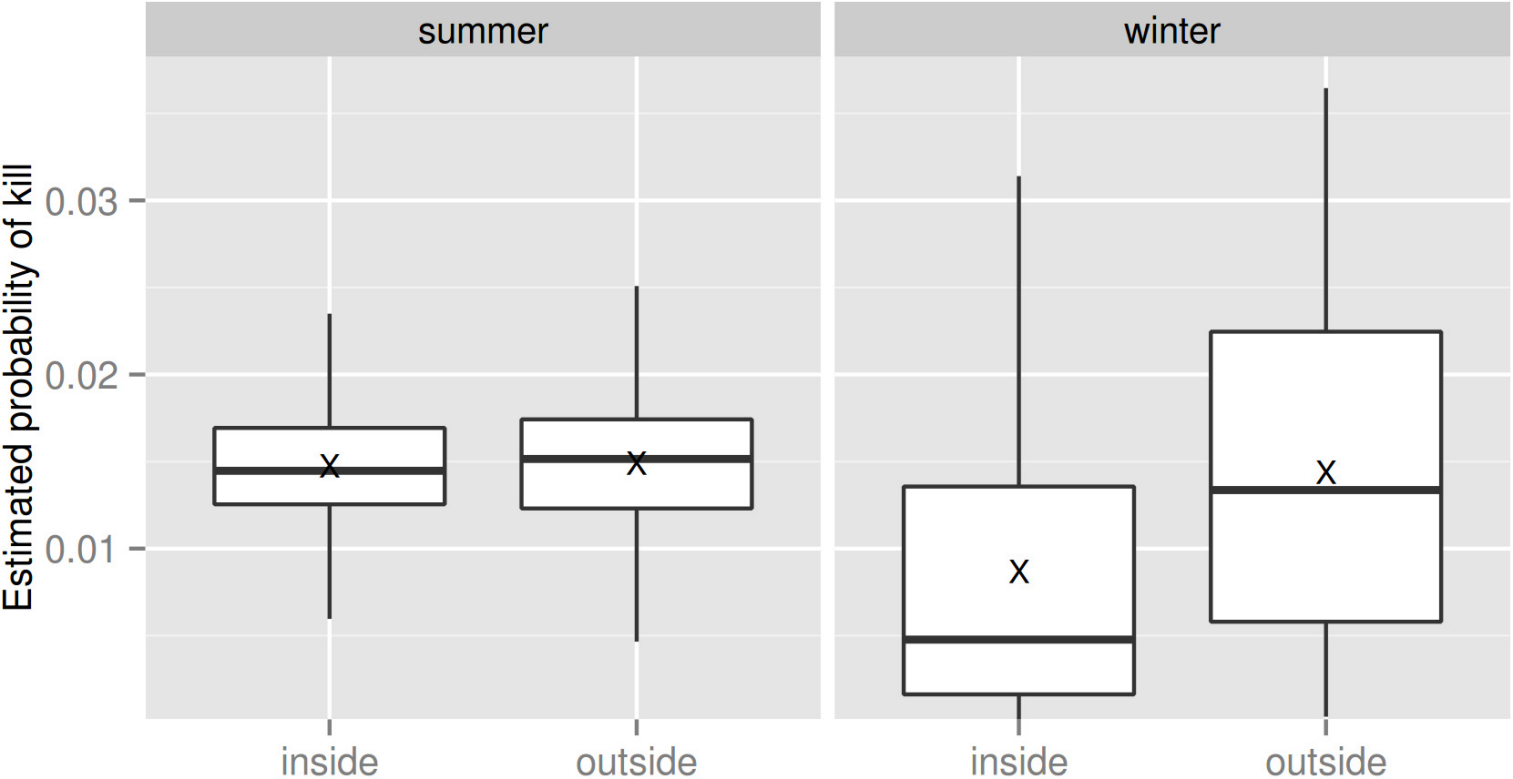
Distribution of the estimated probability of observing at least one deer killed by lynx. Boxplots represent the distribution of the estimated values inside and outside the national parks, during summer and winter respectively. “X” denote the mean values, while the horizontal bold lines denote the median values.

Based on our model, in winter the estimated mean probability of observing at least one killed deer was much higher outside than inside the national parks (0.0144 ± 0.0096 and 0.0087 ± 0.0089, respectively, mean ± s.e., Fig 3), whereas in summer the estimated values were similar in both areas (0.0149 ± 0.0052 outside and 0.0147 ± 0.0045 inside, respectively, mean ± s.e., Fig 3).

**Fig 4:**
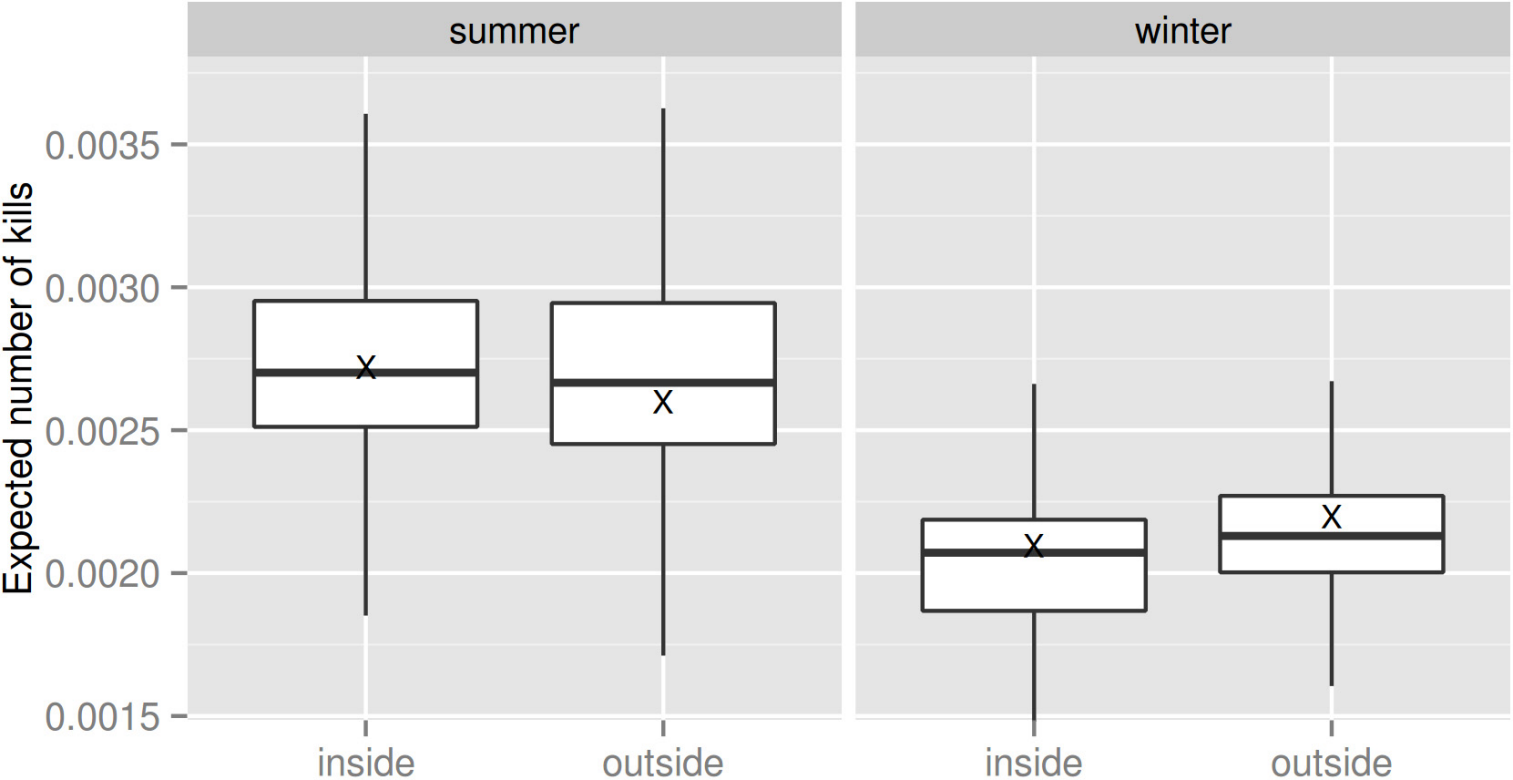
Distribution of the expected number of deer killed per km^2^. Boxplots represent the distribution of the estimated values inside and outside the national parks, during summer and winter respectively. “X” denote the mean values, while the horizontal bold lines denote the median values.

In winter, the estimated expected number of kills per km^2^ was higher outside than inside the national parks (0.0022 ± 0.0003 and 0.0021 ± 0.0003, respectively, mean ± s.e., Fig 4), whereas in summer the opposite situation was observed (0.0026 ± 0.0005 outside and 0.0027 ± 0.0003 inside the national parks, mean ± s.e., Fig 4). Despite being apparently very small, these differences are unitless and should therefore be seen in relationship with their respective ranges.

## Discussion

In accordance with our expectations, our analysis of the spatio-temporal aspect of lynx predation revealed a seasonal shift on the spatial distribution of predation risk, with predation by lynx in winter concentrated outside of the national parks. In summer, the probability to have one or more deer killed by lynx did not differ between areas, but the expected number of kills per area unit was higher inside the national parks than outside. Our results also confirmed that the average roe deer kill rates found in the BFE were higher than those reported for most of Scandinavia, and similar to those found elsewhere in Central Europe and Southern Sweden, but red deer kill rates differed. Finally, kill rates on both deer species were influenced by season and by the reproductive status of female lynx, but not by lynx sex and age nor according to the individual.

Although the annual per-capita kill rates that we determined have to be considered as minimum values, as we may not have recognized all prey killed by the monitored lynx, we are confident that at least in the case of wild ungulate prey, our calculations are very close to reality because (1) the reliability of our method for the identification of potential killed prey has already been tested and demonstrated by Krofel et al. [60] and Ersson [73]; (2) during our study, we also checked about 300 additional single lynx positions in the field (and found prey remains at such locations only once); and (3) for our calculations, we also included “virtual kills” (according to Nilsen et al. [27]). The risk to have killed domestic animals that could not be detected based on GPS-position clusters may be higher, as lynx may abandon killed livestock earlier than wild prey [74]. However, we are confident that the lack of any killed domestic animal in our prey series corresponds well to reality, because remains of domestic prey have never been found in lynx scats from the BFE [44], and according to regional authorities of the regions of Pilsen and South Bohemian (personal communication), only very few requests of compensation for livestock killed by lynx are submitted yearly in the entire BFE. This supports the premise that lynx predation on livestock and the conflicts derived therefrom are minor issues in most European countries [3]. Finally, we acknowledge that monitoring more individuals of each “lynx status” and all animals at the same time would have improved our dataset. Despite this, we consider the overall number of monitored lynx (ten) to be sufficiently representative, given that the number of independent individuals annually documented in the study area ranged between 18 and 23, and that during the study period the level of turnover of resident individuals was low (estimated based on data from 6-year camera trapping monitoring [75]). Moreover, differences between individual lynx as well as between years of monitoring did not seem to have any impact, which leads us to believe that the number of observations is adequate for the type of analyses performed.

### Spatio-temporal aspect of lynx predation

Although Nilsen et al. [27] had already analyzed the variations in lynx kill rates between adjacent areas along a gradient of climatic conditions, to our knowledge our study is the first that explicitly investigated how the patterns of lynx predation vary spatially and temporally and with respect to the presence of protected areas. For wintertime, the observed higher probability of predation and the larger expected number of killed deer per area unit outside the national parks compared to inside corresponded well to the higher winter concentration of deer prey at lower elevations [52]. This is in accordance with the expectation that prey density and spatial distribution will affect the foraging behavior of individual carnivores [76, 77]. However, in the BFE, our previous study, which focused on lynx predation on red deer inside the national parks, showed that lynx in winter were more likely to kill red deer prey in areas with medium to low red deer densities [78]. This suggests that the winter concentration of lynx predation in the foothills, as found in the current study, is not related to the seasonal migration of red deer, but rather of roe deer [41], which is clearly the main prey of lynx during both seasons (e.g., [44]).

Similarly, during summer, the observed lack of substantial differences in probability of predation between the different parts of the study area likely corresponded to a more dispersed deer distribution throughout the entire BFE, resulting from both the seasonal changes in deer grouping behavior [39] and their expansion to higher elevations [37, 38, 40]. On the other hand, the larger number of expected kills per area unit inside the national parks compared to outside in summer may be explained by the lynx selection, at a medium to large spatial scale, of high forest cover [4, 79, 80], large distances from human presence ([80, 81]; but see also [79]), and high densities of ungulate prey [4, 78, 79]. Therefore, in summer, with more homogeneous local ungulate densities, lynx may tend to spend a longer time in areas of high forest cover and far from human presence, i.e. inside the two national parks. In fact, in summer, when not influenced by local variations in prey densities, lynx tended to spend more time in these strictly protected areas (55.6% of lynx GPS-positions were located inside national parks in summer, only 49.6% in winter). This may be because they are safer than the unprotected surroundings, where poaching increases lynx mortality [4]. Therefore, the observed seasonal changes altogether may be considered as a response to the trade-off between abundance of prey and avoidance of human activity (seen as a source of mortality) which has already been described in Scandinavia by Basille et al. [79] and Bunnefeld et al. [81].

### Quantitative aspect of lynx predation: comparing annual kill rates from the BFE with those from other European regions

Overall, the actual annual per-capita predation rates found in our study area barely differed from those found in other Central European areas: 56−72 ungulates per lynx per year in the Swiss Jura Mountains [24] and 55 ungulates per lynx per year in the Swiss Alps [31] and in the Dinaric Mountains [29]. For a better comparison of our results with those of previous studies both in Central Europe and in Scandinavia, we recalculated the prey-specific per-capita annual kill rates, based on the mean prey times for ungulate prey and on the frequency of each prey on the total that are reported in those published studies (according to Krofel et al. [29]).

Throughout Central Europe, the average number of roe deer killed by a single lynx in one year is relatively constant (Table 5), and the slight variations in mean kill rates between areas may be simply due to differences in the representation of each “lynx status” among lynx monitored in each study. Furthermore, differences between areas in the relative importance of alternative prey species may also have an influence. For Southern Norway, Gervasi et al. [14] reported kill rates for roe deer that were less than half of those found in the BFE (Table 5). Although lynx is an efficient predator even at low roe deer densities [27], this is most likely due to the very low density of this deer species in Scandinavia [28], which leads to prey other than roe and red deer (namely semi-domestic reindeer, domestic sheep and hare) playing a more substantial role in the lynx diet [14, 28]. Accordingly, Andrén and Liberg [35] found annual predation rates that were comparable to those from Central Europe in Southern Sweden, in an area with relatively high roe deer density and without other large alternative prey available.

**Table 5:** Comparison of lynx mean roe deer per-capita kill rates from the BFE with those found elsewhere in Europe.

Reference	Study area and status of lynx population	Lynx main prey species	Kill rates as expressed in the reference	Number of followed lynx and their “status”	Mean annual per-capita roe deer kill rates
Breitenmoser and Haller, 1993 [30]	Swiss Alps (expanding lynx population)	Roe deer, chamois	Time between consecutive (ungulate) kills: 5.0 days and 5.1 days in the center and front of the population, respectively. Killed roe deer = 62% and 26% of all killed ungulates in the center and front of the population, respectively	14 lynx monitored (but only 2 males + 2 females intensively monitored: 1male+1female in the population front and 1male+1female in the population centre)	45.42 killed roe deer/365 days in the centre and 18.96 killed roe deer/365 days in the front of the population
Okarma et al., 1997 [23]	Polish part of the Bialowieza Primeval Forest (established lynx population)	Roe deer, red deer	Mean prey time = 5.4 days. Killed roe deer = 74% of all killed deer (62% of all found kills)	11 lynx (including males, single females and females with kittens; not specified in which proportions)	50.02 killed roe deer/365 days.
Jobin et al., 2000 [25]	Swiss Jura Mountains (established lynx population)	Roe deer, chamois	Mean interval between consecutive ungulate kills = 5.9 days for females, 5.2 days for males (mean = 5.55 days) Killed roe deer = 76% of all killed ungulates (69% of all found kills)	29 lynx (including males, single females and females with kittens; not specified in which proportions)	49.99 killed roe deer/365 days
Krofel et al., 2014 [29]	Slovenian Dinaric Mountains (established, declining lynx population)	Roe deer, dormouse	Killed roe deer = 88% of all killed ungulates found by telemetry. 1 roe deer killed every 7.64 days on average	8 collared lynx (5 females + 3males; not reported which females reproduced in which year)	47.78 killed roe deer/365 days
Gervasi et al., 2013 [14]	Southern Norway (established lynx population)	Roe deer, red deer, sheep, (locally reindeer)	4.2 killed roe deer/100 days in summer; 9.4 killed roe deer/100 days in winter (mean = 6.8 roe deer/100 days)	30 lynx (14 females and 16 males; not reported which females reproduced in which year)	24.82 killed roe deer/365 days
Andrén and Liberg, 2015 [35]	Southern Sweden (established lynx population, but locally expanding)	Roe deer (mountain hare, black grouse, capercaillie)	4.85 roe deer/30 days for adult males; 2.71 roe deer/30 days for solitary females; 6.23 roe deer/30 days for family groups (mean = 4.6 roe deer/30 days)	17 lynx (6 adult males, 6 solitary females, 5 family groups)	55.97 killed roe deer/365 days
This study	BFE: Germany, Czech Republic (established lynx population)	Roe deer, red deer	Killed roe deer = 79% of all found kills. Actual annual predation rate = 44.83 roe deer/365 days; adult female = 50.08 roe deer/365 days; 54.46 roe deer/365 days; Family groups = 74.62 roe deer/365 days (Table 4)	10 lynx (4 females and 6 males; all females reproduced)	53.50 killed roe deer/365 days

The mean annual (365 days) per-capita roe deer kill rates were recalculated either based on the reported mean prey time and on the percentage of killed roe deer on the total of killed ungulates or as a mean of values reported for summer and winter.

The mean per-capita annual kill rate of red deer in the BFE (3.86 killed red deer/year) was much lower than the recalculated red deer per-capital annual kill rates from both Poland (26.78 killed red deer/year, recalculated from [23]) and Southern Norway (8.03 red deer/year, recalculated from [14]). This may be due to differences in red deer abundance between areas: although no absolute estimations are available for Southern Norway [14], the estimated red deer densities reported for Poland are higher than those obtained for the BFE (5.01–8.20 roe deer/km^2^ counted in spring [23]). Moreover, in Poland, lynx may lose a red deer carcass when it is found by wolves (e.g., [22]) and overall scavenging pressure seems quite high [82, 83], which may also account for a higher number of red deer killed by lynx. Finally, in the BFE, the presence of enclosures in which a large portion of the red deer population overwinters may also reduce the proportion of red deer killed by lynx, as conditions inside these fenced areas do not seem to be very favorable for lynx when hunting [78]. In accordance with both previous studies, in the BFE, red deer annual kill rates differed widely throughout the year and between lynx belonging to different lynx statuses, with a much higher number of red deer killed in winter than in summer and with adult males killing the highest number of red deer. However, because adult male lynx hold much larger territories than adult female lynx (Table 1, [84]), the mean red deer “annual kill rates per km^2^” were equal for adult males and single adult females. In addition, although our analyses did not confirm any relevant influence of individual lynx on the values of per-capita annual kill rates overall, in the BFE, individual differences in the number of red deer killed by monitored male lynx in a year were evident (Table 1).

### Quantitative and seasonal aspects of lynx predation: factors influencing kill rates

Regarding the effect of “lynx status”, our analyses indicated that family groups had the shortest prey times and thus the highest annual kill rates and the highest predation rates per km^2^, which is in accordance with what was found in other studies across Europe (e.g., [23, 24, 27, 29, 31, 35]). Given that both handling time and searching time likely contributed to this result, the most probable explanation is that predation rates in family groups are determined by the food demands of kittens, and females with kittens cannot spend long periods without any available large prey [23, 81].

Regarding the effect of season, the higher kill rates in winter than in summer found in our study are in contrast with other findings from Central Europe [23, 29] but in accordance with those from Southern Norway [14]. These results may be considered counter-intuitive, as in summer, with higher temperatures leading to quicker meat decay [85], lynx would be expected to abandon the kill earlier. However, the rapid decay of meat may instead result in lynx relying more on smaller prey species in summer than in winter, as actually observed in the BFE [44] and elsewhere in Europe ([21, 23, 28]; but see also [86]). The higher proportion of killed alternative prey may then be one of the factors leading to lower deer kill rates in summer than in winter. Accordingly, Gervasi et al. [14] found that lynx killed more deer in winter than in summer, in an area where grazing sheep were available during summer, whereas Andrén and Liberg [35] found no seasonal differences in an area with no available sheep year-long.

In addition, it is expectable that, in winter, the more clumped and predictable prey distribution [38, 40] will improve prey detectability [27], while limited food availability and snow cover will increase deer vulnerability [87–89]. Both factors likely reduce searching time, but we found that the shorter winter prey time was mainly related to a reduction in handling time. Nevertheless, our “searching time” not only consisted of the time lynx spent hunting, but also included territory patrolling and looking for mating partners. As winter comprises lynx mating season, the longer time dedicated to these activities during this period [90] likely counteracted the effects of reduced hunting time on winter “searching time”.

The higher mobility during mating season could also push both male and female lynx to abandon partially unconsumed prey items, contributing to the observed shorter winter handling time. In addition, in the case of family groups, during the first winter months (until kittens abandon their mother), energy demands increase as kittens mature [25], likely shortening their winter handling time. In summer, during the natal season females with newborn kittens are forced to limit their movements and maximally exploit each killed prey [91], likely prolonging their summer handling time.

Seasonal differences in scavenging pressure may also play a role: in winter, lynx tracks in snow may increase the detectability of non-decaying carcasses [82] and food scarcity may induce red fox and wild boar to rely more on scavenging [83, 92]. Such species are abundant on the Czech side of the BFE [93], feed at lynx kills [25] and wild boars are able to completely consume them during a single scavenging visit [22]. However, an experimental study in the Bavarian Forest National Park [85] suggested a rather modest rate of vertebrate scavenging at ungulate carcasses in the BFE.

Finally, winter handling time may be reduced by the difficulty in obtaining meat of killed prey (as it quickly freezes at the constantly low ambient temperatures [94]) and processing frozen meat in the stomach [95]. This is likely one of the causes of the seasonal differences in kill rates observed in Scandinavia [14] and may have contributed to our results, as in the BFE the temperature is permanently below 0°C during 40−70 days/year [96].

### Actual and perceived impact of lynx predation and conflicts with hunters

In order to quantify the actual impact of lynx predation on the entire red and roe deer populations, at the scale of the study area, i.e., hundreds to thousands of km^2^, our estimated values of per-capita actual annual kill rates would have to be combined with data on the number of lynx of each “lynx status” living in the entire BFE, and the resulting values would have to be compared with reliable absolute estimations of roe and red deer abundance or survival and reproduction rates (e.g., [35]). Unfortunately, such data are not available for the entire BFE, therefore we can only infer the extent to which lynx is likely to limit deer abundances. However, the perceived local impact of lynx predation, at the scale of the hunting ground, i.e., 0.5 to tens km^2^, can likely influence the lynx−hunter conflicts, and thus the level of poaching, more strongly than the actual overall impact of predation. Consequently, the attitude of hunters towards lynx may become more negative in those areas where lynx predation is locally more intense [18]. To evaluate such local intensity, it is necessary to consider the values of per-capita “annual kill rates per km^2^” of the different lynx statuses in combination with each other. In fact, in established lynx populations, the territory of a male overlaps with that of one or more females, which can be either accompanied by their kittens or not [84]. Furthermore, subadult lynx, that do not hold their own territories, generally float and hunt between the home ranges of resident individuals [84].

The extremely low annual per-capita predation rates of red deer suggested that in the BFE, as elsewhere in Europe, predation by lynx is unlikely to be a limiting factor for the population growth of this prey species [23, 97]. With regard to the local impact, even in areas that are used by an adult male, an adult female and a subadult lynx at the same time the total annual predation would be well below 0.1 red deer/km^2^. This value represents less than 14% of the annual red deer recruitment estimated for the Bavarian Forest National Park (0.7 calves/km^2^ in 2009; Bavarian Forest National Park Administration, unpublished data).

Regarding roe deer, recent studies suggest that predation by lynx is mostly additive to other mortality [35, 98] and has a greater impact in regions with lower vegetation productivity and harsh winters [99]. In the BFE, Heurich et al. [100] found that predation by lynx was the main cause of mortality for GPS-collared roe deer inside the Bavarian Forest National Park, where this species is not hunted and its density reaches the minimum value for the BFE [52]. On the other hand, in the Czech foothills and Šumava Protected Landscape Area, roe deer hunting bags increased yearly between 1997 and 2003, reaching levels comparable to those before lynx reintroduction, and seemed relatively stable from 2004 to 2013 (source: Czech Forest Management Institute– ÚHUL), which suggests that the roe deer population has not declined substantially during the last two decades, despite the presence of the lynx. Based on our results, the local impact of lynx predation in the BFE may vary from a minimum of 0.52 roe deer killed annually per km^2^ in areas where the territory of a male lynx overlaps with that of a single adult female (i.e., 0.11 plus 0.41 roe deer respectively, killed annually per km^2^), to a maximum of 1.17 roe deer killed annually per km^2^ in areas that are at the same time inhabited by an adult male lynx, a family group and a floating subadult (i.e., 0.11 plus 0.61 plus 0.45 roe deer killed annually per km^2^). In comparison, in Bavaria (Germany) in 2009, hunters shot 1.19 roe deer/km^2^ in state-managed hunting grounds where environmental characteristics are similar to those of the Bavarian Forest National Park, whereas they shot 3.84 roe deer/km^2^ in hunting grounds located in the Bavarian foothills (source: Bavarian State Ministry for Food, Agriculture and Forestry). On the Czech side of the BFE, in the hunting grounds adjacent to the Šumava National Park and regularly frequented by monitored lynx, including the Protected Landscape Area and the unprotected surroundings, hunters in 2009 shot about 2 roe deer per km^2^ per year, ranging from 1.17 to 4.35 roe deer per km^2^ per year, with the highest annual hunting bags being recorded for hunting grounds located in the unprotected part of the BFE (source: Czech Forest Management Institute- ÚHUL).

These data indicated that, in most of the unprotected foothills, the number of roe deer annually shot by hunters per km^2^ is two to three times higher than the maximum number of roe deer killed by lynx per km^2^(as calculated based on our models and on the spatial organization and social structure of lynx population [84]). Therefore, one may expect that lynx−hunter conflicts due to predation on roe deer will be limited. This may be true at least during summer, when the spatial distribution of lynx predation is relatively uniform, per-capita kill rates for lynx of all statuses are lower than the corresponding mean annual values, and the largest expected number of kills per unit area are found inside the two national parks. However, our results depict a substantially different situation in winter, when the spatial distribution of lynx predation is less homogeneous, per-capita kill rates for lynx of all statuses are higher than the corresponding mean annual values, and the largest expected number of kills per unit area is found outside of the two national parks. As a consequence, the local impact of lynx predation is likely to become higher in winter in this less-protected part of the BFE, where sport hunting is practiced. As is evident from Fig 2B, most of the areas where winter predation concentrates on the Czech side are located even beyond the borders of the Šumava Protected Landscape Area, which serves as a buffer zone in which forestry and hunting are practiced in the traditional way, but hunters and foresters communicate and cooperate with conservation biologists. Especially in the unprotected part of the BFE, on both sides of the national border, a local higher intensity of lynx predation may actually lead to an exacerbation of lynx−hunter conflicts, which may contribute to an increased poaching level in the proximity of protected areas [4].

## Conclusions

In summary, our results from the summer months supported the conclusions of Müller et al. [4], that at least large protected areas in Europe are important source areas for lynx populations that minimally endow the territories of several individuals with safety zones. On the other hand, our results from the winter months indicated that even the largest protected areas may not ensure sufficient protection to lynx if the migration routes and wintering areas of its prey species lie beyond the boundaries of such areas (see also [43]), and even the individuals forming the core population are exposed to the risk of poaching when they follow their prey.

Because illegal killings in the surroundings of protected areas can seriously hinder the expansion of large carnivores and reduce connectivity among populations (e.g., [4]), which may even jeopardize the conservation efforts undertaken within the borders of protected areas [101], based on our empirical data, we suggest that unprotected, multi-use landscapes of the BFE should be given the focus in future lynx conservation efforts. Given the size and characteristics of most European protected areas (e.g., [7, 43]), this recommendation likely applies also to other European regions that host populations of large carnivores. In practice, we propose that managers focusing on the conservation of large carnivores should place more effort in identifying potential conflict zones not only within but also outside of protected areas. To achieve this, scientific information about the predation patterns of a given predator species should be collected, considering the quantitative, seasonal, and spatio-temporal aspects at the same time. Such relevant information should also be used to mitigate conflicts with hunters.

In the case of the lynx, our results can be extrapolated also to several other areas throughout Central Europe. To mitigate lynx-hunters conflicts in the multi-use landscapes surrounding protected areas, we propose to (a) define a common “wildlife management unit” comprising both summer and winter ranges of deer (i.e. both protected areas and a wide buffer of unprotected surroundings); (b) establish local wildlife management working groups, including conservation biologists, foresters and hunters, in order to improve coordination and communication between stakeholders; and (c) use scientific data to broaden the discussion from the point of view of the local, “perceived impact” of predation, to that of the overall, “actual impact” of predation. Finally, we recommend that roe deer should not be shot inside National Parks, as good local densities of this prey species may increase the attractiveness of protected areas for the lynx and thus limit predation impact in the adjacent unprotected hunting grounds.

## Supporting Information

S1 Appendix Modeling procedure for the two-stage predation risk model. (DOCX)

S1 Dataset Prey dataset including all found and virtual kills by GPS-collared lynx in the BFE. (XLSX)

S1 Table Summer and winter predicted prey time, handling time, searching time and predation rates with bootstrap-percentile-intervals for each “lynx status”. S.E. = standard error. (DOCX)
