# Supplementary material for: Patterns of Lynx Predation at the Interface between Protected Areas and Multi-Use Landscapes in Central Europe
Source: PLoS One. 2015 Sep 17;10(9):e0138139. doi: 10.1371/journal.pone.0138139 (PMC4574974; doi:10.1371/journal.pone.0138139)
Supplement: S1 Appendix — (DOCX) [file pone.0138139.s001.docx]

**S1 Appendix. Modeling procedure for the two-stage predation risk model**

The spatial distribution of predation events was modeled as a landscape of fear [1], based on the aggregated number of deer kills within a study area rasterized into 6,740 500 x 500m quadrants. Our goal was to obtain an estimation of the expected number of kills for all quadrants located in the study area, including those where no kill had been observed. A common challenge when working with spatial data is that these are often zero-inflated, i.e. a large majority of the observation points contain no data/zero counts. The technical implication when modeling such data is that the distributional assumptions (e.g. the Poisson distribution for count data) are violated. We observed kills in only 144 (2.14%) quadrants for the data pertaining to the winter months (October to March) and in 189 (2.80%) quadrants for the summer months.

The kill observations were matched to lynx individuals for time periods in which their movements were tracked using GPS and VHF telemetry data. Because of differences between the individuals in terms of length of their monitoring period and number of readings, the raw kill counts pertaining to each lynx individual were weighted according to the time period in which they were radio marked. While the raw data are discrete counts, these standardized kill data are on a continuous scale, but limited to be ≥0 – the zero-inflation remains a challenge though.

To deal with the zero-inflation, we utilized a two-stage generalized additive model [2]. With$Y_{i}$ being the number of kills in quadrant $i$ and $Z_{i}$ the binary information whether one or more kills had been observed within that quadrant, we wished to model the expected number of kills in each quadrant$i$, . This may be written as follows, where the expected number of kills () in a quadrant is expressed conditional on whether one or more kills take place or not, multiplied by the probabilities () that each event will occur:

This simplified representation of , in which is the probability to have at least one kill, leads us to the two stage model.

**Stage 1 – Modeling the probability of observing at least one kill**

In stage 1, we use the information whether one or more kills were observed within each quadrant $i$, with $Z_{i}\text{=1}$ where one or more kills were observed and $Z_{i}\text{=0}$ where no kills were observed. We assumed a binomial distribution, i.e.

$$Z_{i}\in\left\{0,1\right\}$$

$p\in\left[0,1\right]$,

where $p$ is the probability of one or more kills to be observed. As it is impossible to determine whether an observed zero in a quadrant is caused by the fact that no kill has taken (or could take) place in that quadrant or by the fact that the observation is simply missing, we needed to perform a spatial smoothing of the available data. This was done by modeling the probability of observing one or more observations within a quadrant,$\hat{p}_{i}$, using a logistic regression model with the response function

where we included the geographic coordinates along with other biotic and abiotic factors characterizing each quadrant into the linear predictors $\text{η}_{i}^{\left(1\right)}$. The index (1) denotes the first model stage.

**Stage 2 – Modeling the expected number of kills**

In stage 2, the number of (standardized) kills was modeled. As for stage 1, it is not possible to determine the reason for observing no kills and stage 2 thus only makes use of information from the quadrants in which at least one kill was observed, i.e. where$Z_{i}\text{=1}$. We assumed a Gamma distribution for the expected number of kills, $Y_{i}$, with

$$Y_{i}\sim Gamma\left(\text{κ,θ}\right)$$

$$Y_{i}\in\text{]0,}\infty\text{[}$$

and shape and scale parameters

The expected number of kills in quadrants in which one or more kills were observed was thus modeled using a generalized additive model with the response function

in whose linear predictors $\text{η}_{i}^{\left(2\right)}$ geographic coordinates and other biotic and abiotic factors were included.

**Combining both model steps**

Combining both models, for all quadrants, we first estimated the probability to have at least one kill made by lynx in each of them. We then multiplied these probabilities with the estimated number of kills from stage 2.

**References**

- 1. Laundré JW, Hernández L, Ripple WJ. The landscape of fear: ecological implications of being afraid. Open Ecology Journal 2010; 3:1-7.
  2. Wood SN. Generalized Additive Models: An Introduction with R. Chapman & Hall/CRC, Boca Raton; 2006. ISBN 978-1584884743.
