# Supplementary material for: Patterns of Lynx Predation at the Interface between Protected Areas and Multi-Use Landscapes in Central Europe
Source: PLoS One. 2015 Sep 17;10(9):e0138139. doi: 10.1371/journal.pone.0138139 (PMC4574974; doi:10.1371/journal.pone.0138139)
Supplement: S1 Table — (DOCX) [file pone.0138139.s003.docx]

S1 Table. Summer and winter predicted prey time, handling time, searching time and predation rates with bootstrap-percentile-intervals for each “lynx status”. S.E. = standard error.

| Lynx status | Prey species |  | Prey time [days ± S.E.] | Handling time [days ± S.E.] | Searching time [days ± S.E.] |  | Predicted predation rate (95% confidence interval) | | |
| --- | --- | --- | --- | --- | --- | --- | --- | --- | --- |
|  |  |  |  |  |  |  | **Lower bound** | **Estimate** | **Upper bound** |
| Summer | | | | | | | | | |
| Adult male | Roe deer |  | 6.30 ± 0.31 | 3.58 ± 0.18 | 2.67 ± 0.41 |  | 19 | 25 | 31 |
| Adult female | Roe deer |  | 7.38 ± 0.64 | 4.39 ± 0.38 | 2.99 ± 0.80 |  | 16 | 22 | 28 |
| Subadult female | Roe deer |  | 6.77 ± 0.59 | 3.86 ± 0.34 | 3.23 ± 0.87 |  | 18 | 24 | 29 |
| Family group | Roe deer |  | 5.02 ± 0.38 | 2.96 ± 0.18 | 1.36 ± 0.32 |  | 25 | 32 | 38 |
|  |  |  |  |  |  |  |  |  |  |
| Adult male | Red deer |  | 10.58 ± 0.91 | 5.96 ± 0.51 | 5.37 ± 1.43 |  | 10 | 15 | 20 |
| Adult female | Red deer |  | 12.38 ± 1.54 | 7.32 ± 0.91 | 6.01 ± 2.32 |  | 9 | 13 | 17 |
| Subadult female | Red deer |  | 11.36 ± 1.42 | 6.44 ± 0.81 | 6.50 ± 2.52 |  | 10 | 14 | 19 |
| Family group | Red deer |  | 8.42 ± 0.99 | 4.94 ± 0.58 | 2.73 ± 1.00 |  | 14 | 19 | 25 |
|  |  |  |  |  |  |  |  |  |  |
| Winter | | | | | | | | | |
| Adult male | Roe deer |  | 5.07 ± 0.34 | 2.74 ± 0.19 | 2.04 ± 0.42 |  | 25 | 31 | 38 |
| Adult female | Roe deer |  | 5.93 ± 0.53 | 3.37 ± 0.30 | 2.29 ± 0.63 |  | 20 | 27 | 34 |
| Subadult female | Roe deer |  | 5.44 ± 0.53 | 2.96 ± 0.29 | 2.47 ± 0.75 |  | 23 | 30 | 36 |
| Family group | Roe deer |  | 4.03 ± 0.35 | 2.27 ± 0.20 | 1.04 ± 0.28 |  | 32 | 40 | 47 |
|  |  |  |  |  |  |  |  |  |  |
| Adult male | Red deer |  | 8.50 ± 0.71 | 4.58 ± 0.38 | 4.11 ± 1.07 |  | 13 | 18 | 23 |
| Adult female | Red deer |  | 9.95 ± 1.16 | 5.62 ± 0.65 | 4.60 ± 1.67 |  | 11 | 16 | 21 |
| Subadult female | Red deer |  | 9.13 ± 1.13 | 4.94 ± 0.62 | 4.97 ± 1.91 |  | 13 | 18 | 23 |
| Family group | Red deer |  | 6.77 ± 0.78 | 3.79 ± 0.44 | 2.09 ± 0.75 |  | 18 | 24 | 29 |
